# Bilateral occlusive retinal vasculitis secondary to intravitreal faricimab injection: a case report and review of literature

**DOI:** 10.1186/s40662-024-00416-y

**Published:** 2024-12-03

**Authors:** Yong Min Lee, Rajya Gurung, Jagjit Singh Gilhotra, Sumu Simon, Sudha Cugati

**Affiliations:** 1 Ophthalmology Department, Royal Adelaide Hospital, Port Road, Adelaide, SA 5000 Australia; 2 Ophthalmology Department, Modbury Hospital, Adelaide, SA 5069 Australia; 3 Faculty of Health and Medical Sciences, The University of Adelaide, Adelaide, SA 5000 Australia

**Keywords:** Occlusive vasculitis, Faricimab, Intraocular inflammation, Intravitreal injection, Age-related macular degeneration

## Abstract

**Background:**

This article describes a rare occurrence of bilateral retinal occlusive vasculitis secondary to intravitreal faricimab injection.

**Case presentation:**

A 72-year-old female with age-related macular degeneration presented with bilateral retinal occlusive vasculitis following intravitreal faricimab injections. The patient was treated with 3 days of intravenous methylprednisolone followed by oral prednisolone taper and topical steroid therapy. Resolution of retinal occlusive vasculitis was observed 2 months post treatment.

**Conclusions:**

Retinal occlusive vasculitis is a rare complication of intravitreal anti-vascular endothelial growth factor (anti-VEGF), particularly with faricimab injections. We also present a review of literature regarding retinal occlusive vasculitis following intravitreal anti-VEGF injections and propose further information regarding its pathophysiology.

## Background

Age-related macular degeneration (AMD) is the leading cause of vision loss in the elderly population in developed countries [1]. Optical coherence tomography (OCT) assists ophthalmologists by identifying key pathological features of AMD and monitoring disease activity in response to treatment [2]. Anti-vascular endothelial growth factor (anti-VEGF) plays an important role in managing angiogenic and inflammatory retinal conditions. Faricimab is a bispecific immunoglobulin G (IgG) monoclonal antibody that inhibits both vascular endothelial growth factor-A (VEGF-A) and angiopoietin-2 (Ang-2) [3]. It was approved by the Therapeutic Goods Administration in Australia in August 2022 for the management of neovascular AMD and diabetic macular edema (DME) [4].

Faricimab is hypothesised to have superior durability and function compared to anti-VEGF monotherapy as shown in the TENAYA and LUCERNE phase 3 clinical trials [3]. These studies demonstrated that nearly 80% of patients treated with intravitreal (IV) faricimab maintained 12-week or 16-week dosing intervals, while achieving non-inferior outcomes to aflibercept in terms of mean change in best-corrected visual acuity (BCVA) from baseline at 48 weeks [3]. The extended dosing intervals can also enhance patient compliance by reducing the treatment burden.

Large clinical trials have demonstrated that the rates of intraocular inflammation (IOI) were low across both faricimab and aflibercept groups [3, 5]. However, the YOSEMITE and RHINE phase 3 clinical trials indicated a numerically higher incidence of IOI in the faricimab group with 17 patients experiencing IOI compared with four patients in the aflibercept group [5]. Although most events were of mild to moderate severity, characterised by anterior chamber inflammatory activity, there were two cases of severe vitritis in the faricimab group that required treatment withdrawal [5]. Similarly, the TENAYA and LUCERNE studies noted a greater number of IOI cases in the faricimab group although the overall rates were comparable [3]. Mild to moderate uveitis occurred in 13 patients receiving faricimab compared to eight patients receiving aflibercept [3]. The TRUCKEE study reported an incidence of 0.53% for serious IOI in its cohort of 376 eyes, with one case of anterior uveitis occurring after the fourth injection [6]. Severe IOI following IV faricimab has been reported, with three cases showing severe sterile inflammation of the posterior segment [7]. To the best of our knowledge, two cases of retinal occlusive vasculitis have been reported following faricimab injection [8, 9].

## Case presentation

A 72-year-old female presented with bilateral ocular pain following IV faricimab injections. She was diagnosed with neovascular AMD and received IV aflibercept injections since 2019 in her right and 2021 for her left eye. The patient’s past medical history included hypertension and hypercholesterolaemia, for which she was treated with olmesartan, amlodipine, hydrochlorothiazide, and fenofibrate. These systemic medications are not associated with retinal vasculitis. There was no history of other autoimmune diseases or prior episodes of uveitis.

The patient had received 18 IV aflibercept injections in her right eye and 16 in her left eye. Due to the recurrence of subretinal fluid upon extending the treatment intervals, she was switched to bilateral IV faricimab injections (10-week intervals for the right eye and 5-week intervals for the left eye). She had received one dose of IV faricimab in the right eye and three doses in the left eye whilst maintaining a BCVA of 20/25 bilaterally and showing no signs of inflammatory activity.

**Fig. 1:**
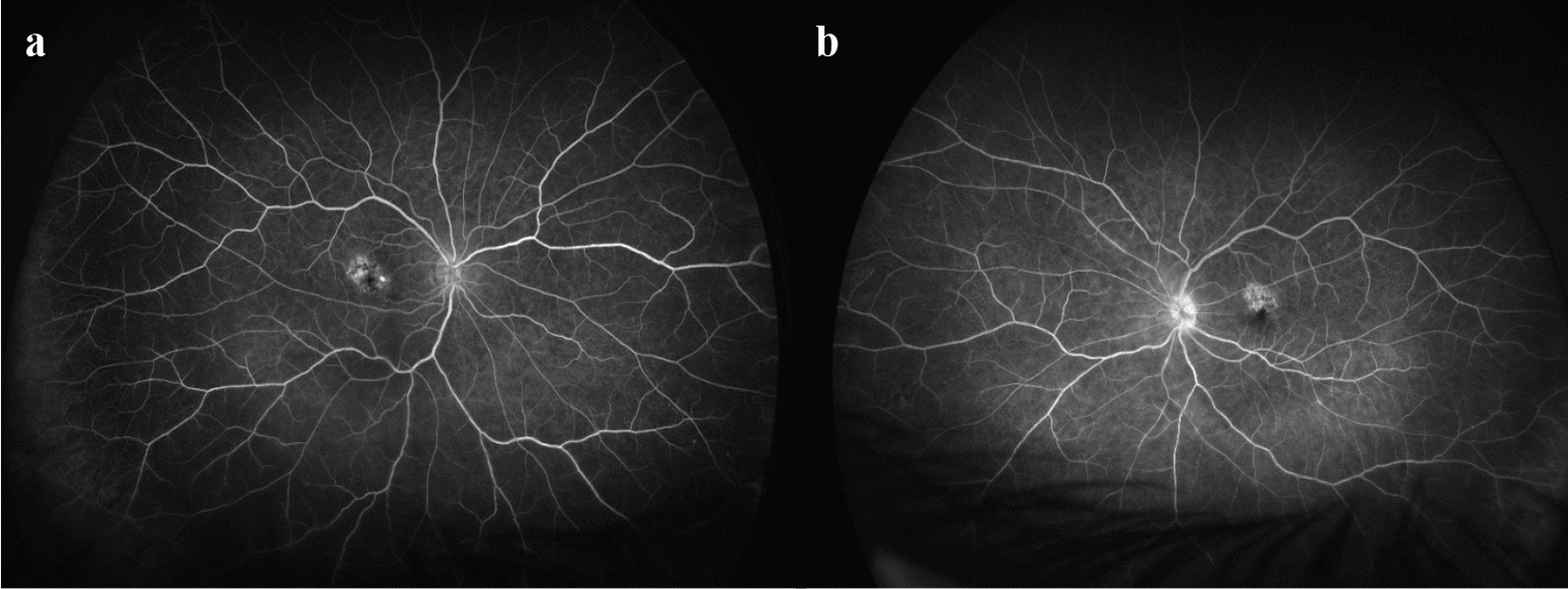
FFA of the right and left eye on initial presentation with bilateral anterior uveitis. **a** Good arterial filling of the right eye with hyperfluorescent leakage in the macular region consistent with neovascular AMD; **b** Good arterial filling of the left eye with similar hyperfluorescent leakage in the macular region consistent with neovascular AMD. FFA, fundus fluorescein angiography; AMD, age-related macular degeneration

The patient presented 13 days after receiving her 3rd IV faricimab injection in the left eye with bilateral red eyes. Her last IV faricimab for the right eye was administered 7 weeks prior. On presentation, her BCVA was 20/25 in both eyes, with intraocular pressures of 30 mmHg and 26 mmHg in her right and left eye, respectively. Slit-lamp examination demonstrated 1 + anterior chamber cells in her right eye and 3 + anterior chamber cells with keratic precipitates in the left eye. Examination was consistent with bilateral anterior uveitis with no evidence of posterior segment involvement. Fundus fluorescein angiography demonstrated no vascular leakage (Fig. 1a and b). Initial serum screening demonstrated weak positive serology for rheumatoid factor (RF), but negative results for interferon gamma release assay (IGRA), herpes simplex virus (HSV), varicella zoster virus (VZV) immunoglobulin M (IgM) serology, normal angiotensin converting enzyme (ACE), and antinuclear antibody (ANA) levels. Chest X-ray demonstrated an inflammatory consolidation that was further investigated with a computed tomography (CT) of the chest that showed bilateral parenchymal scarring with moderate to severe emphysema. The patient was prescribed topical prednisolone-phenylephrine (1%–0.12%) four times daily for the right eye and hourly for the left eye, hydrocortisone acetate 1% eye ointment at night and twice daily topical timolol 0.5% for both eyes. Three weeks after her initial presentation, the patient showed improvement in her symptoms whilst on gradual tapering of topical prednisolone-phenylephrine 1%–0.12% (twice daily on the right eye and six times a day on her left eye). Her BCVA was 20/25 and 20/20 in her right and left eye. Slit lamp examination demonstrated quiet anterior chamber activity in the right eye and 1 + anterior chamber cell activity in the left eye with old pigmented keratic precipitates. Considering the bilateral presentation of uveitis and the fact that the most recent IV faricimab in her right eye was given 7 weeks ago, it was concluded that the anterior uveitis was likely unrelated to faricimab injections. After discussing the risks and benefits of continuing anti-VEGF treatment, she was retreated with bilateral IV faricimab injections.

**Fig. 2:**
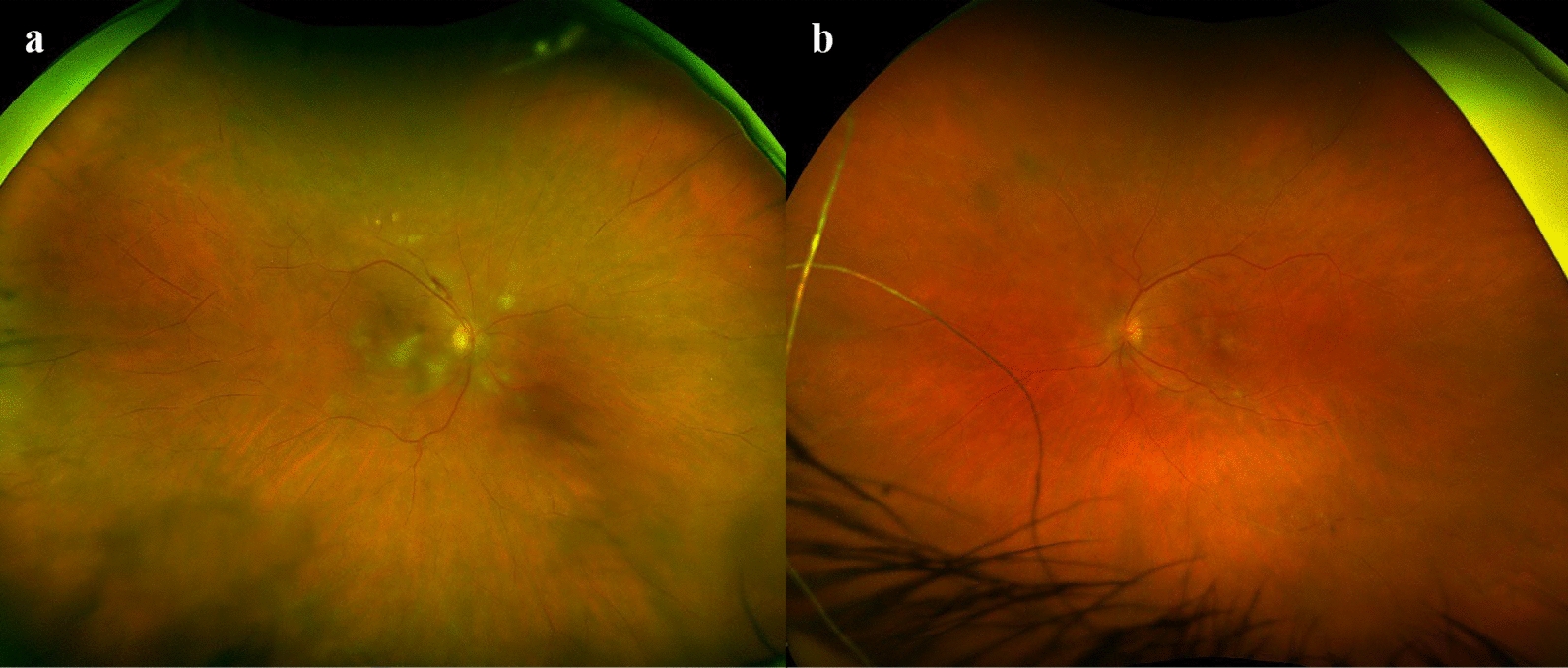
Fundus photo of both right and left eye on presentation 11 days following her bilateral intravitreal faricimab injections. **a** Fundus photo of the right eye showing peripapillary cotton wool spots with extensive arteritis and arteriolar whitening in the peripheries. There is perivascular haemorrhage along the superior arcade vessels; **b** Fundus photo of the left eye showing cotton wool spots along the inferior arcade vessels and perivascular whitening in the peripheries consistent with arteritis

**Fig. 3:**
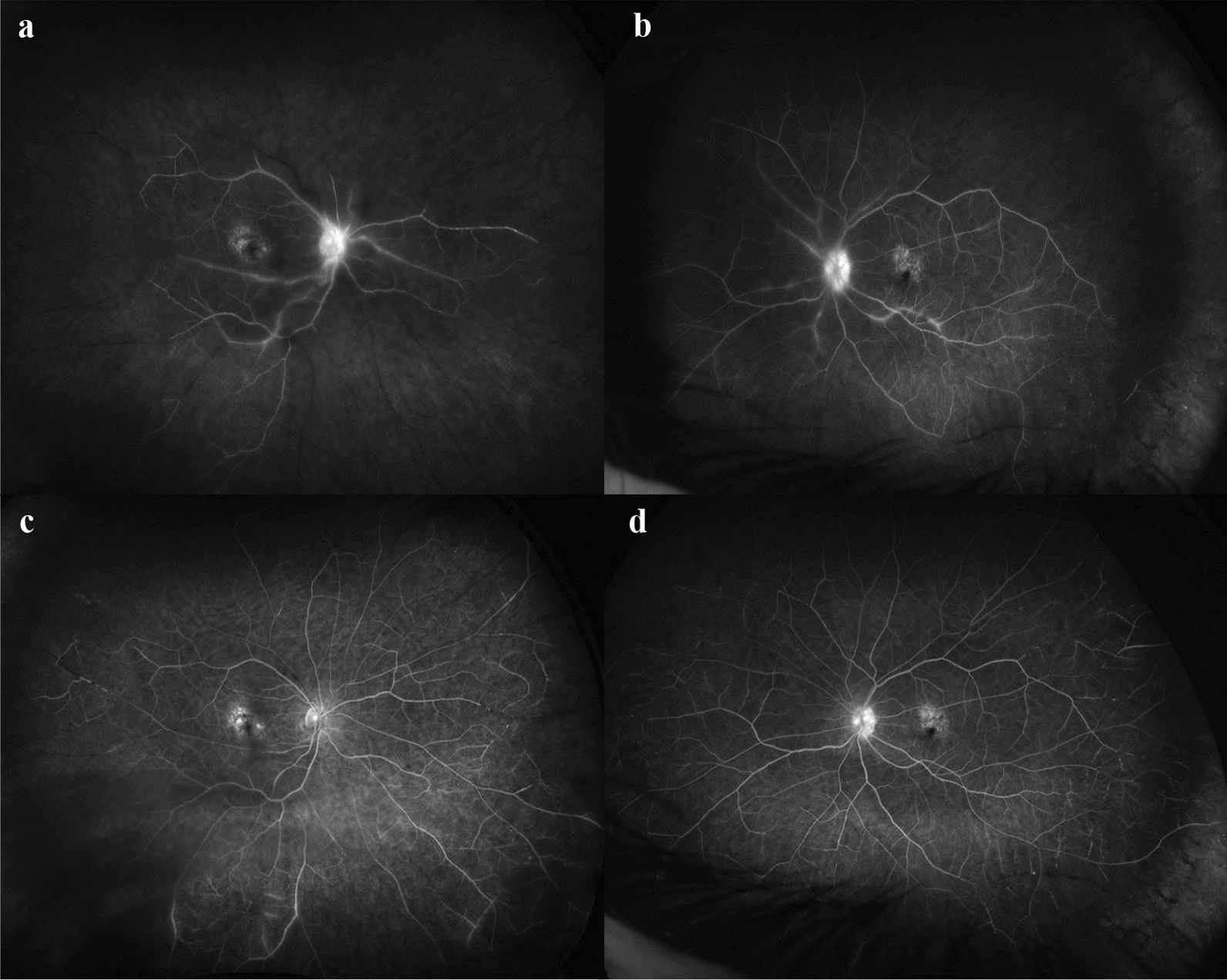
FFA on presentation to emergency and 2 months following treatment with systemic steroid therapy. **a** FFA of the right eye 11 days following its second intravitreal faricimab injections showed delayed vascular filling consistent with occlusive vasculitis; **b** FFA of the left eye 11 days following its fourth intravitreal faricimab injections also showed delayed vascular filling consistent with occlusive vasculitis (right eye more severe than left eye); **c** FFA of the right eye at 2-month follow-up showed delayed but improved filling of the vessels and resolved perivascular leakage; **d** FFA of the left eye at 2-month follow-up showing similar resolved perivascular leakage. FFA, fundus fluorescein angiography

The patient re-presented to the emergency department 11 days following her bilateral IV faricimab injections with blurry vision in the right eye associated with left temporal headaches. She had received a total of two IV faricimab injections in her right eye and four injections in her left eye prior to presentation. BCVA was 20/80 and 20/25 in the right and left eye, respectively, and intraocular pressures were 14 mmHg bilaterally. She was still administering topical prednisolone-phenylephrine to the left eye six times a day (she had ceased all other therapy at this time). Anterior segment examination demonstrated mild anterior chamber inflammation with 0.5 + cells and keratic precipitates, and 2 + cells in the anterior vitreous in both eyes. Dilated fundus examination showed bilateral disc swelling, cotton wool spots, perivascular sheathing, and haemorrhages (see Fig. 2a and b). OCT demonstrated bilateral drusen with pigment epithelium detachment that appeared stable. Fluorescein angiography demonstrated an ischaemic peripheral retina with bilateral occlusive vasculitis (right more severely affected, see Fig. 3a and b).

Serum infective and inflammatory screens were positive for Epstein-Barr virus (EBV) IgM serology, RF (weakly positive) and anti-neutrophil cytoplasmic antibody (ANCA) levels. Serum testing returned negative for Neisseria meningitidis, streptococcus pneumoniae, syphilis, tuberculosis, toxoplasmosis, HSV, VZV, enterovirus, ACE, anti-double stranded deoxyribonucleic acid, ANA, anti-myeloperoxidase (MPO) antibody, beta-2 glycoprotein IgG antibody, anti-cyclic citrullinated peptide (anti-CCP), myelin oligodendrocyte glycoprotein (MOG) antibody, neuromyelitis optic (NMO) antibody and human leukocyte antigens (HLA) B51. Vitreous aspirate was performed on the right eye with negative microscopy, cultures and sensitivity (MCS) and polymerase chain reaction (PCR) for infective organisms. CT angiography of the head and neck, magnetic resonance imaging (MRI) and MRI angiogram of the brain and orbits revealed no significant carotid artery arterial stenosis, occlusion or features of systemic vasculitis.

A diagnosis of bilateral occlusive vasculitis secondary to faricimab was made and the patient was treated with intravenous methylprednisolone for three days followed by a tapering regimen of oral prednisolone from 50 mg. Topical prednisolone-phenylephrine (1%–0.12%) was increased to every 2 h bilaterally. After consultation with stroke and vascular teams, she was started on daily aspirin 100 mg and clopidogrel 75 mg. The patient improved with BCVA of 20/40 and 20/25 in her right and left eye, respectively, at the 2-month follow-up. There was resolution of cotton wool spots and perivascular haemorrhage and sheathing (Fig. 3c and d). The patient was observed for 4 months following discharge without receiving IV anti-VEGF injections. Her right eye remained dry, but there was an increase in subretinal fluid in her left eye with a BCVA of 20/32 and 20/25 in her right and left eye, respectively. No cells were detected in her anterior and posterior chambers bilaterally, and the patient had discontinued both topical and systemic steroids therapy 2 months prior. The patient agreed to recommence IV aflibercept treatment for the left eye every 4 weeks whilst continuing to observe the right eye.

## Discussion

Retinal occlusive vasculitis is a rare complication following IV anti-VEGF injections. A comprehensive literature review was conducted on PubMed and Embase from January 1, 1970 to March 28, 2024. The search aimed to identify articles related to retinal occlusive vasculitis following IV injections using the keywords “retinal vasculitis” and “intravitreal injection”, “aflibercept”, “ranibizumab”, “bevacizumab”, “brolucizumab”. A total of 447 articles were identified, of which 137 duplicates were removed. Title, abstract and full-text screening were completed by YML on COVIDENCE, and 18 relevant articles available in the English were included. Four other articles were found through references and reviewer recommendations which were also included (see Table 1).

**Table 1:** Cases of occlusive retinal vasculitis following IVI

Author	Study design	Year	Number of cases	Age	Sex	Laterality	Indication	IVI agent	Number of injections	Other IVI	Onset	Initial VA	Treatment regime	Final VA
Baxter et al. [12]	Case report	2015	1	83	F	Right	BRVO	Ranibizumab	2	Nil	16 h	HM	Vitrectomy: 2 IV antibiotics PO prednisolone Laser therapy Gas tamponade Sub triamcinolone	20/200 (4 w)
Jain et al. [19]	Case report	2020	1	92	F	Left	AMD	Brolucizumab	3	Multiple bevacizumab, ranibizumab, aflibercept	16 d	CF	Top prednisolone	–
Haug et al. [16]	Case report	2020	2	88	F	Both	AMD	Brolucizumab	1	Right: 26 ranibizumab Left: 21 ranibizumab	4 w	Right: 20/40 Left: 20/50	Top prednisolone IV dexamethasone	–
Baumal et al. [11]	Retrospective case series	2020	15	77.6	F: 12	–	AMD	Brolucizumab	1.4	27.5	30.3 d	20/191	Top prednisolone Top difluprednate PO valacyclovir IV solumedrol	20/136 (25 d)
Iyer et al. [18]	Case report	2020	1	76	F	Right	AMD	Ranibizumab	3	Multiple bevacizumab, ranibizumab, aflibercept	1 w	20/200	Top prednisolone PO methylprednisolone Vitrectomy IV triamcinolone	–
Witkin et al. [23]	Retrospective case series	2020	26	79.1	F: 22 M: 3	Right: 13 Left: 13	–	Brolucizumab	1 injection: 11 2 injections: 11 3 injections: 4	39	26 d	20/151	Top steroids: 24 PO steroids: 11 IV steroids: 5 Vitrectomy: 4 Observation: 2 IV antibiotics: 1 Antiviral: 2	20/243 (53 d)
Mones et al. [22]	Post hoc analysis	2021	23	–	–	–	AMD	Brolucizumab	–	–	25.5 d	–	–	–
Takada et al. [27]	Case report	2021	1	77	M	Right	AMD	Brolucizumab	1	19 ranibizumab 33 aflibercept	2 m	–	Top betamethasone	–
Walter et al. [28]	Retrospective case series	2021	1	–	–	–	AMD	Brolucizumab	–	–	–	–	–	–
Aziz et al. [10]	Retrospective chart review	2021	1	–	–	–	AMD	Brolucizumab	–	–	–	–	–	–
Khanani et al. [6]	Randomised double-masked, phase 3A study	2022	7	–	–	–	AMD	Brolucizumab	–	–	–	–	–	–
Kusuhara et al. [21]	Case report	2022	1	75	F	Right	AMD	Brolucizumab	1	1 aflibercept	3 w	20/400	Top betamethasone PO prednisolone PO kallidinogenase PO aspirin	20/25 (5 w)
Kim et al. [26]	Retrospective chart review	2023	1	71	M	Left	AMD	Brolucizumab	1	6 ranibizumab 25 aflibercept	6 w	CF	PO prednisolone Top prednisolone Sub triamcinolone PO DAPT	20/200 (3 w)
Zubrikcy et al. [25]	Retrospective chart review	2023	2	–	–	–	AMD	Brolucizumab	–	–	–	–	–	–
Tadayoni et al. [29]	Multicentered prospective single-arm study	2023	4	–	–	–	AMD	Brolucizumab	–	–	–	–	–	–
Wykoff et al. [24]	Phase 3, double-masked, active-controlled randomised clinical trial	2023	7	–	–	–	DME	Brolucizumab	–	–	–	–	–	–
			2	–	–	–	DME	Aflibercept	–	–	–	–	–	–
Hirano et al. [17]	Case report	2023	1	68	M	Right	DME	Brolucizumab	3	–	1 m	20/32	Top betamethasone Sub triamcinolone Top brimonidine Top dorzolamide	–
Bodaghi et al. [13]	Prospective phase IIIb single arm multicenter studies	2023	2	–	–	–	–	Brolucizumab	–	–	–	–	–	–
Gillies et al. [14]	Retrospective chart review	2023	2	–	–	–	–	Brolucizumab	–	–	–	–	–	–
Grewal et al. [15]	Retrospective case series	2024	9	–	–	–	AMD	Brolucizumab	–	–	–	–	–	–
Li et al. [8]	Case report	2024	2	96	F	Both	AMD	Faricimab	1	Right: 15 ranibizumab, 38 aflibercept Left: 21 aflibercept	18 d	Right: 20/160 Left: CF	Top dexamethasone Top brinzolamide PO prednisolone	Right: 20/160 (7 w) Left: CF (7 w)
Chen et al. [9]	Case report	2024	1	73	M	Left	PCV	Faricimab	2	0	2 w	20/50	IV dexamethasone	–

*IVI* = intravitreal injections; *VA* = visual acuity; *F* = female; *M* = male; *BRVO* = branch retinal vein occlusion; *AMD* = age-related macular degeneration; *DME* = diabetic macular oedema; *h* = hour; *d* = day; *w* = week; *m* = month; *HM* = hand motion; *CF* = counting fingers; *IV* = intravitreal; *PO* = oral; *Sub* = subtenon; *Top* = topical; *DAPT* = dual-antiplatelet therapy; *PCV* = polypoidal choroidal vasculopathy

A total of 113 cases have been identified in the literature across 22 studies [8–29]. The average age of affected patients was 79.3 years. The most frequently associated agent was IV brolucizumab, which was implicated in 106 cases (93.8%). Case reports of retinal occlusive vasculitis have been documented following ranibizumab (2), aflibercept (2) and faricimab (3). The average number of injections administered before the onset of retinal occlusive vasculitis was 1.62 and the mean time to symptom development following the last injection was 26.1 days. There is no consensus on the optimal management for retinal occlusive vasculitis following IV anti-VEGF injections; however, treatments have included topical, subtenon, oral and IV steroids, IV antibiotics, antivirals and vitrectomy.

Acute onset IOI following IV injections typically occurs within the first few days and often presents as anterior chamber inflammation. Its incidence has been reported to be as high as 19% following IV injections [30]. Delayed onset IOI following IV anti-VEGF injections has been reported, particularly in association with brolucizumab. Retinal occlusive vasculitis has features of both types III and IV hypersensitivity. However, a recent pathological examination of a vitreous sample from a patient with retinal vasculitis secondary to brolucizumab revealed a predominance of T cells, suggesting a type IV hypersensitivity [18, 30].

The literature suggests an association between retinal vasculitis and systemic inflammatory conditions such as rheumatoid arthritis, Wegener’s granulomatosis, microscopic polyangiitis and Churg-Strauss syndrome [31]. Antibody-antigen interactions with inflammatory markers like RF or ANCA can cause structural changes in neutrophil adhesive molecules, leading to dysfunctional attachment to vascular endothelial cells. The release of proteolytic enzymes, reactive oxygen species, complement activation can result in progressive inflammatory damage and necrosis of the vascular wall [32]. Ocular involvement of ANCA-associated vasculitis more commonly includes peripheral ulcerative keratitis, scleritis or uveitis [33]. Retinovascular involvement is rare, with a retrospective study involving 1286 patients with systemic necrotising vasculitis reporting only five cases of retinal vasculitis, most associated with Wegener’s granulomatosis [34].

Brolucizumab has been reported as an anti-VEGF agent with higher rates of IOI, attributed to its specific pharmacological properties. The HAWK and HARRIER trials demonstrated that inflammatory vasculitis occurred in 3.3% of brolucizumab injections, with 2.1% of these cases being retinal occlusive vasculitis [35]. Similarly, the OCTOPUS and SWIFT studies reported that the rate of IOI in anti-VEGF naive patients was 10.5%, with 1.4% of cases involving vascular occlusion with or without vasculitis [13]. A higher anti-drug antibody level to brolucizumab, ranging from 35%–52% in treatment-naive patients, compared to less than 5% for aflibercept or ranibizumab, may predispose brolucizumab to trigger an inflammatory response [3, 36]. Brolucizumab also has a relatively smaller molecular size, allowing for higher molar concentration and increased tissue penetration, potentially increasing its exposure to the immune system [30]. This theoretically enables brolucizumab to have greater therapeutic effects, particularly for conditions like AMD where the blood-retina barrier is compromised, but it also increases the likelihood of IOI [30].

Patient factors that predispose individuals to IOI post IV anti-VEGF injections include naturally higher levels of anti-drug antibodies against the anti-VEGF agent, retinal comorbidities such as AMD with a dysfunctional blood-retina barrier, and other pro-inflammatory conditions like uveitis [30]. Anti-VEGF medications are manufactured using recombination gene technology with bacteria or animal cells and the presence of foreign cells, along with endotoxin contamination, can contribute to inflammatory responses following IV injections [30]. The delivery and preparation of anti-VEGF injections may also contribute to triggering an IOI response. Silicone oil is applied to the syringes to reduce plunger resistance, but it can be injected into the eye with the medication [37]. Silicone oil can induce aggregation of immunogenic protein complexes, and the rate of its release can be influenced by external factors such as temperature and light exposure [37]. With the incidence of three cases of severe sterile posterior chamber inflammation secondary to faricimab injections within a 1-month time frame at three different clinics and two different lot numbers, manufacturing and storage issues that may contribute to IOI also needs to be considered [7]. Our case involved IV faricimab injections from different batches for both eyes.

Faricimab is a bispecific antibody with characteristics that may predispose to inflammatory responses. The presence of the Fc antibody portion of the anti-VEGF has been associated with higher rates of IOI, likely due to increased expression of Fc receptors in AMD [30]. Aflibercept and bevacizumab had higher rates of IOI than ranibizumab, which had an absence of the Fc antibody molecule [30]. The preparation of faricimab involves physical agitation by the physician to remove excess air from the solution. Physical stimulus can increase the rate of silicone oil release during the injection that may induce immune complex formation [38]. A case-controlled retrospective study reported that all patients that received IV anti-VEGF after being flicked had a higher IOI compared to those that did not have such physical stimulus [37].

## Conclusion

We report a rare case of bilateral retinal occlusive vasculitis secondary to faricimab injections. With the increasing use of IV injections in the management of vitreoretinal diseases such as neovascular AMD and DME, clinicians should be aware of the risks and recognise the clinical features of occlusive vasculitis following IV faricimab injection. Administration of IV anti-VEGF in the presence of mild anterior chamber inflammatory activity should warrant caution due to the risk of sensitisation. It is possible that the patient developed acute-onset sterile inflammation and sensitisation in response to faricimab at her initial presentation. Further elicitation with a repeated dose of faricimab resulted in a type IV hypersensitivity resulting in occlusive vasculitis. The onset of retinal occlusive vasculitis in this case was consistent with the two previous cases following IV faricimab injections at roughly 2 weeks although both cases did not have any active inflammation at the time of injection [8, 9]. Chen et al. describes a similar experience where this pathology occurred after an initial injection that likely led to sensitisation [9]. The treatment response to high-dose steroid therapy further supports the immune mediated mechanism in this case. Larger studies are needed to explore the mechanisms of retinal occlusive vasculitis and to develop effective treatment regimens for this complication.

## Highlights

- Case of bilateral retinal occlusive vasculitis (ROV) following intravitreal faricimab injections.
- Review of literature of ROV following intravitreal anti-vascular endothelial growth factor (anti-VEGF) injections.
- ROV following intravitreal anti-VEGF injections is likely a type IV hypersensitivity reaction.
- Our case presents fundus and fundus fluorescein angiography images implicated in this pathology.

## Data Availability

All data generated or analysed during this study are included in this published article.

## References

[1] Vyawahare H, Shinde P. Age-related macular degeneration: epidemiology, pathophysiology, diagnosis, and treatment. Cureus. 2022;14(9): e29583. 36312607 10.7759/cureus.29583 PMC9595233

[2] Bhende M, Shetty S, Parthasarathy MK, Ramya S. Optical coherence tomography: a guide to interpretation of common macular diseases. Indian J Ophthalmol. 2018;66(1):20–35. 29283118 10.4103/ijo.IJO_902_17 PMC5778576

[3] Heier JS, Khanani AM, Quezada Ruiz C, Basu K, Ferrone PJ, Brittain C, et al. Efficacy, durability, and safety of intravitreal faricimab up to every 16 weeks for neovascular age-related macular degeneration (TENAYA and LUCERNE): two randomised, double-masked, phase 3, non-inferiority trials. Lancet. 2022;399(10326):729–40. 35085502 10.1016/S0140-6736(22)00010-1

[4] Vabysmo Australian Government | Department of Health and Aged Care | Therapeutic Goods Administration. https://www.tga.gov.au/resources/auspmd/vabysmo.

[5] Wykoff CC, Abreu F, Adamis AP, Basu K, Eichenbaum DA, Haskova Z, et al. Efficacy, durability, and safety of intravitreal faricimab with extended dosing up to every 16 weeks in patients with diabetic macular oedema (YOSEMITE and RHINE): two randomised, double-masked, phase 3 trials. Lancet. 2022;399(10326):741–55. 35085503 10.1016/S0140-6736(22)00018-6

[6] Khanani AM, Aziz AA, Khan H, Gupta A, Mojumder O, Saulebayeva A, et al. The real-world efficacy and safety of faricimab in neovascular age-related macular degeneration: the TRUCKEE study - 6 month results. Eye (Lond). 2023;37(17):3574–81. 37173428 10.1038/s41433-023-02553-5 PMC10686385

[7] Thangamathesvaran L, Kong J, Bressler SB, Singh M, Wenick AS, Scott AW, et al. Severe intraocular inflammation following intravitreal faricimab. JAMA Ophthalmol. 2024;142(4):365–70. 38421861 10.1001/jamaophthalmol.2024.0530 PMC10905372

[8] Li Y, Chong R, Fung AT. Association of occlusive retinal vasculitis with intravitreal faricimab. JAMA Ophthalmol. 2024;142:489. 38517431 10.1001/jamaophthalmol.2024.0928

[9] Chen X, Wang X, Li X. Intra-ocular inflammation and occlusive retinal vasculitis following intravitreal injections of faricimab: a case report. Ocul Immunol Inflamm. 2024. 10.1080/09273948.2024.2361834. 38856728 10.1080/09273948.2024.2361834

[10] Aziz AA, Khanani AM, London N, Hagen MM, Danzig CJ, Kulkarni AD, et al. Real world efficacy and safety of brolucizumab in neovascular AMD: the REBEL study. Invest Ophthalmol Vis Sci. 2021;62(8):451.

[11] Baumal CR, Spaide RF, Vajzovic L, Freund KB, Walter SD, John V, et al. Retinal vasculitis and intraocular inflammation after intravitreal injection of brolucizumab. Ophthalmology. 2020;127(10):1345–59. 32344075 10.1016/j.ophtha.2020.04.017

[12] Baxter KR, Robinson JE, Ruby AJ. Occlusive vasculitis due to hyperacute *Streptococcus mitis* endophthalmitis after intravitreal ranibizumab. Retin Cases Brief Rep. 2015;9(3):201–4. 25764316 10.1097/ICB.0000000000000138

[13] Bodaghi B, Souied EH, Tadayoni R, Weber M, Ponthieux A, Kodjikian L. Detection and management of intraocular inflammation after brolucizumab treatment for neovascular age-related macular degeneration. Ophthalmol Retina. 2023;7(10):879–91. 37343623 10.1016/j.oret.2023.06.009

[14] Gillies M, Dang T, Nguyen V, Invernizzi A, Romano F, Cozzi M, et al. Six-month outcomes of brolucizumab in routine clinical practice: data from the fight retinal blindness! registry. Invest Ophthalmol Vis Sci. 2023;64(8):466.

[15] Grewal DS, Wykoff CC, D’Souza D, Jehl V, Alecu I, Jaffe GJ. Imaging features of retinal vasculitis and/or retinal vascular occlusion after brolucizumab treatment in the postmarketing setting. Ophthalmol Sci. 2024;4(1): 100361. 37869023 10.1016/j.xops.2023.100361 PMC10587630

[16] Haug SJ, Hien DL, Uludag G, Ngoc TTT, Lajevardi S, Halim MS, et al. Retinal arterial occlusive vasculitis following intravitreal brolucizumab administration. Am J Ophthalmol Case Rep. 2020;18: 100680. 32258827 10.1016/j.ajoc.2020.100680 PMC7125319

[17] Hirano T, Toriyama Y, Takahashi Y, Hoshiyama K, Murata T. Retinal arterial occlusive vasculitis after multiple intravitreal brolucizumab injections for diabetic macular edema. Am J Ophthalmol Case Rep. 2023;29: 101788. 36632338 10.1016/j.ajoc.2022.101788 PMC9826871

[18] Iyer PG, Peden MC, Suner IJ, Patel N, Dubovy SR, Albini TA. Brolucizumab-related retinal vasculitis with exacerbation following ranibizumab retreatment:a clinicopathologic case study. Am J Ophthalmol Case Rep. 2020;20: 100989. 33294727 10.1016/j.ajoc.2020.100989 PMC7695942

[19] Jain A, Chea S, Matsumiya W, Halim MS, Yasar C, Kuang G, et al. Severe vision loss secondary to retinal arteriolar occlusions after multiple intravitreal brolucizumab administrations. Am J Ophthalmol Case Rep. 2020;18: 100687. 32280811 10.1016/j.ajoc.2020.100687 PMC7139151

[20] Khanani AM, Brown DM, Jaffe GJ, Wykoff CC, Adiguzel E, Wong R, et al. MERLIN: Phase 3a, multicenter, randomized, double-masked trial of brolucizumab in participants with neovascular age-related macular degeneration and persistent retinal fluid. Ophthalmology. 2022;129(9):974–85. 35537533 10.1016/j.ophtha.2022.04.028

[21] Kusuhara S, Kim KW, Miki A, Nakamura M. Angiographic findings before and after the onset of brolucizumab-associated retinal vascular occlusion and intraocular inflammation. Am J Ophthalmol Case Rep. 2022;26: 101521. 35464682 10.1016/j.ajoc.2022.101521 PMC9026644

[22] Mones J, Srivastava SK, Jaffe GJ, Tadayoni R, Albini TA, Kaiser PK, et al. Risk of inflammation, retinal vasculitis, and retinal occlusion-related events with brolucizumab: post hoc review of HAWK and HARRIER. Ophthalmology. 2021;128(7):1050–9. 33207259 10.1016/j.ophtha.2020.11.011

[23] Witkin AJ, Hahn P, Murray TG, Arevalo JF, Blinder KJ, Choudhry N, et al. Occlusive retinal vasculitis following intravitreal brolucizumab. J Vitreoretin Dis. 2020;4(4):269–79. 32789284 10.1177/2474126420930863 PMC7418897

[24] Wykoff CC, Garweg JG, Regillo C, Souied E, Wolf S, Dhoot DS, et al. KESTREL and KITE Phase 3 studies: 100-week results with brolucizumab in patients with diabetic macular edema. Am J Ophthalmol. 2023;260:70–83. 37460036 10.1016/j.ajo.2023.07.012

[25] Zubricky R, McCoy J, Donkor R, Miller DG, Sonbolian N, Heaney A, et al. Real-world frequency and management of ocular adverse events in eyes with neovascular age-related macular degeneration treated with brolucizumab. Ophthalmol Ther. 2023;12(5):2397–408. 37310683 10.1007/s40123-023-00741-w PMC10442012

[26] Kim DJ, Jin KW, Han JM, Lee SH, Park YS, Lee JY, et al. Short-term safety and efficacy of intravitreal brolucizumab injections for neovascular age-related macular degeneration: a multicenter retrospective real-world study. Ophthalmologica. 2023;246(3–4):192–202. 36720210 10.1159/000529410

[27] Fukushima A. Intraocular inflammation-induced visual field defect after an intravitreal brolucizumab injection. EURETINA. 2021.

[28] Walter SD, Saba NJ. Real-world efficacy and safety of brolucizumab. Invest Ophthalmol Vis Sci. 2021;62(8):456.

[29] Tadayoni R, Souied E, Creuzot-Garcher C, Cohen SY, Kodjikian L, Baillif S, et al. Disease control at week 16 of brolucizumab in adult patients with suboptimal anatomically controlled neovascular age related macular degeneration – the SWIFT study. Invest Ophthalmol Vis Sci. 2023;64(8):465.

[30] Anderson WJ, da Cruz NFS, Lima LH, Emerson GG, Rodrigues EB, Melo GB. Mechanisms of sterile inflammation after intravitreal injection of antiangiogenic drugs: a narrative review. Int J Retina Vitreous. 2021;7(1):37. 33962696 10.1186/s40942-021-00307-7 PMC8103589

[31] Matsuo T, Koyama T, Morimoto N, Umezu H, Matsuo N. Retinal vasculitis as a complication of rheumatoid arthritis. Ophthalmologica. 2010;201(4):196–200. 10.1159/000310151 2077456

[32] Macarie SS, Kadar A. Eye involvement in ANCA positive vasculitis. Rom J Ophthalmol. 2020;64(1):3–7. 32292850 PMC7141924

[33] İnanç Tekin M, Çakar Özdal MP. Ophthalmic manifestations of ANCA-associated vasculitis. Acta Medica. 2021;52(4):257–63.

[34] Rothschild PR, Pagnoux C, Seror R, Brézin AP, Delair E, Guillevin L. Ophthalmologic manifestations of systemic necrotizing vasculitides at diagnosis: a retrospective study of 1286 patients and review of the literature. Semin Arthritis Rheum. 2013;42(5):507–14. 23270762 10.1016/j.semarthrit.2012.08.003

[35] Dugel PU, Koh A, Ogura Y, Jaffe GJ, Schmidt-Erfurth U, Brown DM, et al. HAWK and HARRIER: phase 3, multicenter, randomized, double-masked trials of brolucizumab for neovascular age-related macular degeneration. Ophthalmology. 2020;127(1):72–84. 30986442 10.1016/j.ophtha.2019.04.017

[36] Sharma A, Kumar N, Parachuri N, Singh S, Bandello F, Regillo CD, et al. Understanding retinal vasculitis associated with brolucizumab: complex pathophysiology or Occam’s Razor? Ocul Immunol Inflamm. 2022;30(6):1508–10. 34014141 10.1080/09273948.2021.1897628 PMC10919544

[37] Melo GB, Figueira ACM, Batista FAH, Filho A, Rodrigues EB, Belfort R Jr, et al. Inflammatory reaction after aflibercept intravitreal injections associated with silicone oil droplets released from syringes: a case-control study. Ophthalmic Surg Lasers Imaging Retina. 2019;50(5):288–94. 31100159 10.3928/23258160-20190503-05

[38] Uchino T, Miyazaki Y, Yamazaki T, Kagawa Y. Immunogenicity of protein aggregates of a monoclonal antibody generated by forced shaking stress with siliconized and nonsiliconized syringes in BALB/c mice. J Pharm Pharmacol. 2017;69(10):1341–51. 28639328 10.1111/jphp.12765

